# Supplementary material for: Insights into cannabinoid receptor 2 (CB2) anterograde trafficking and pharmacological chaperoning
Source: Cell Mol Life Sci. 2025 Nov 22;83(1):11. doi: 10.1007/s00018-025-05961-w (PMC12748375; doi:10.1007/s00018-025-05961-w)
Supplement: Supplementary file 1 — Supplementary Material 1 (PDF 191 KB) [file 18_2025_5961_MOESM1_ESM.pdf]

Insights into Cannabinoid Receptor 2 (CB2) anterograde trafficking and pharmacological chaperoning

Caitlin RM Oyagawa [1,2], Braden Woodhouse [1,2,3], Karren C Wood [1,2], Michelle Glass [1,2,4,5], Natasha L Grimsey * [1,2,5]

[1] Department of Pharmacology and Clinical Pharmacology, School of Medical Sciences, Faculty of Medical and Health Sciences, University of Auckland, Auckland, New Zealand

[2] Centre for Brain Research, Faculty of Medical and Health Sciences, University of Auckland, Auckland, New Zealand

[3] Department of Oncology, School of Medical Sciences, Faculty of Medical and Health Sciences, University of Auckland, Auckland, New Zealand

[4] Department of Pharmacology and Toxicology, School of Biomedical Sciences, University of Otago, Dunedin, New Zealand

[5] Maurice Wilkins Centre for Molecular Biodiscovery, New Zealand

* Correspondence: n.grimsey@auckland.ac.nz (N L Grimsey)

Supplementary Figures

Supplementary Fig. 1

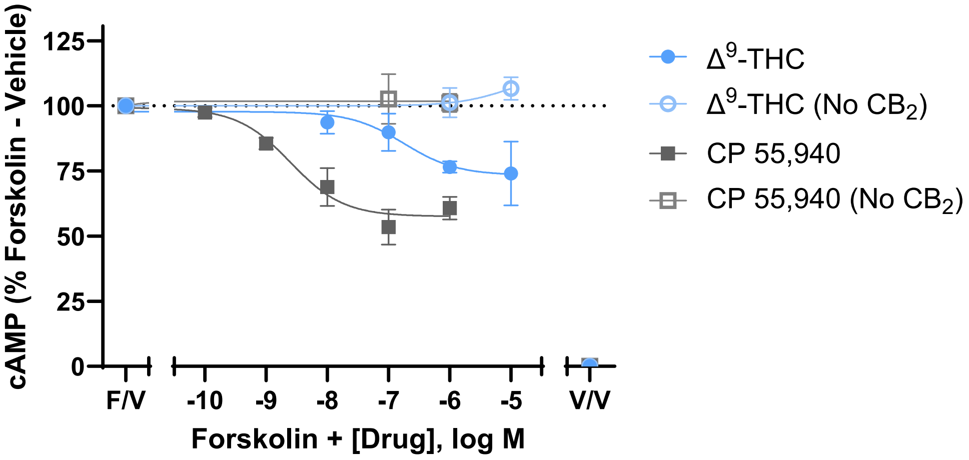

Supplementary Fig. 1 CB$_2$ wt cAMP response to Δ^9-THC and CP 55,940

Cells stably expressing CB$_2$ wt (or untransfected cells, “No CB$_2$”) were treated with forksolin (5 μM) and a dilution series of Δ^9-THC or CP 55,940. Cyclic AMP (cAMP) responses were measured as the mean BRET ratio from the CAMYEL biosensor during a 10 minute stimulation, then normalised to forskolin with vehicle (100%; “F/V”) and vehicle-only control (0%, “V/V”). Data are presented as mean ± SEM from three independent experiments.

Supplementary Fig. 2

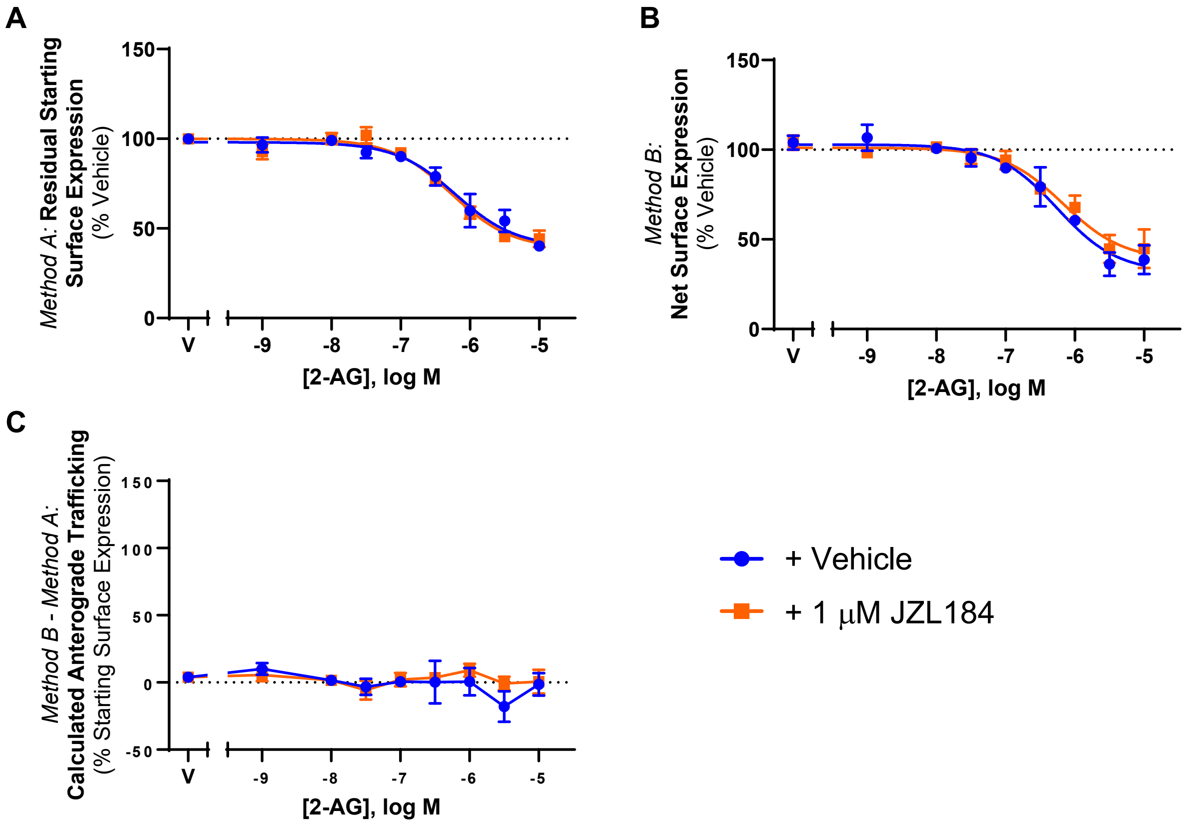

Supplementary Fig. 2 CB_2 wt trafficking in response to 2-AG +/- MAGL inhibitor, JZL184

Cells stably expressing CB_2 wt were treated with a dilution series of 2-AG +/- 1 μM JZL184 for 3 hours, and were labelled to measure (A) residual starting surface expression (*Method A*) or (B) net surface expression (*Method B*). (C) Calculated anterograde trafficking (*Method B – Method A*). Data are presented as mean ± SEM from three independent experiments.
